# The impact of social participation trajectories on depression incidence among Chinese older adults

**DOI:** 10.1186/s12877-025-05924-7

**Published:** 2026-07-20

**Authors:** Yan Liu

**Affiliations:** 1 Present Address: Chengdu Open University, Chengdu, China; 2 School of Ethnology and Sociology, Minzu University of China, 27 Zhongguancun South Aveue, Beijing, 100081 China

**Keywords:** Older adults, Social participation trajectories, Depression

## Abstract

Social participation is a critical factor influencing depression in older adults. This study investigates the impact of social participation trajectories on depression risk among older Chinese adults, using data from five waves of the China Health and Retirement Longitudinal Study (CHARLS, 2011–2020). A life course perspective identified three trajectories of social participation: increasing, low-steady, and decreasing. Cox proportional hazards models revealed that, compared to the low levels of social participation, higher levels of social participation are associated with a lower risk of depression. These findings highlight the protective role of the extent of social participation to mitigate depression risk. This study advances prior research by applying the growth mixture modeling (GMM) approach to capture the temporal dynamics of social participation, offering new support for gerontological theories. Practical implications include improving community infrastructure and expanding childcare and older adult care services to support social participation to prevent depression in later life.

## Clinical trial number

Not applicable.

## Introduction

Geriatric depression, the most common mental disorder after dementia, significantly impacts the quality of life of older adults due to its high morbidity and disability rates [1, 2]. In China, 53.02% of adults over 60 experienced depressive symptoms in 2018, with this trend continuing to rise in recent years [3, 4]. Reducing depression risk among older adults has become a priority amid the challenges of population aging [5].

Social participation, as a key factor affecting depression in older adults, deserves attention. It is described as a person’s involvement in activities that provide interaction with others in society or the community [6], social participation, as a major pillar of active aging [7], can promote a sense of belonging, which in turn has positive effects on physical and mental health [8]. It can also buffer the negative health impacts associated with stress [9]. Whether the risk of depression increases later in life currently has no definitive answer. The morbidity of depressive symptoms in the general population is lowest in middle age and then increases in later adulthood, reaching the highest level among adults aged > 80 years [10]. As one of the major factors influencing depression incidence, participating in social activities can prevent the occurrence of depression [11, 12].

However, treating social participation as static in most of the previous studies may have obscured important variability in the relationship between social participation and health outcomes. This study examined the trajectories of social participation over time and how they influence the incidence of depression, which can provide a more comprehensive understanding of the role of social participation in the incidence of depression in these longitudinal contexts.

## Theoretical background

### Social integration and social participation

In the field of sociology, the study of social participation in individual health can be traced back to Durkheim’s classic study on suicide, which showed that people lacking social ties were less socially integrated and more likely to engage in pathological personal behaviors and deviate from social norms and obligations [13]. As research progresses, the social integration model conceptualized by Berkman et al. provides perspectives on how social participation affects health by outlining different pathways through which social integration impacts health [14]. Social relationships and social integration formed through social participation can influence health not only through biological pathways, such as neuroendocrine reactivity and reduced allostatic load [15] but also through social pathways, such as increasing available resources, buffering the stress brought about by adverse events, and promoting the flow of health-related information [16, 17]. Based on this social integration theory, the social benefit hypothesis states that higher levels of social participation are associated with better health conditions.

Social participation among older adults may be especially beneficial for health. Over the life course, individuals enter and exit multiple social roles, but as they age, they are more likely to lose formal social roles [18]. With the decline of formal social roles, participating in social activities as social participation may become a more important source of social integration.

### Life course and social participation

In theory, the loss of formal roles after retirement results in fewer opportunities for older adults to interact with society. Thus, older adults need more informal social participation activities to maintain connections with society. However, in reality, the heterogeneity of the older population leads to considerable variability in social participation. From a life course perspective, on the one hand, activity choices earlier in life can alter the risk and severity of activity restriction later in life; on the other hand, suppression of midlife social activities may amplify desires for social activities in old age [19].

Three theories in gerontology can provide insights into trends in the dynamic changes in social participation among older adults. First, activity theory posits that social participation increases with age because retired older adults have more leisure time to devote to social participation. Older adults adapt to changing social environments by redefining their social roles and engaging in new and different activities. In contrast, disengagement theory states that social participation generally declines over the life course, accelerating from midlife onward as age increases. This decrease in social participation is natural and stems from increased morbidity in the individual and loss of family and friends. Continuity theory, on the other hand, argues that social participation is consistent over time and is primarily influenced by earlier life stages due to consistent tendencies and motivations at the individual level [20]. As individuals age, they attempt to maintain the roles and activity patterns established earlier in life [21]. For example, older adults continue participating in activities they engaged in during early life. This may explain the strong influence that past activity importance has on the likelihood of participating in that activity [22]. In summary, we can derive three possible trends in social participation trajectories from the above theories: increasing, decreasing, and low-steady. Whether and how much one participates in activities are critical for psychological well-being in older adults [23].

### Social participation and depression

The main mechanisms through which social participation benefits depression are psychosocial pathways [24]. First, participating in social activities helps older adults maintain health and find social and spiritual support, thereby reducing mental health problems. Social participation provides older adults with social support from informal social networks (i.e., relationships with other community members and peers), which in turn benefits their emotional functioning. Second, the potential links between social participation and individual health can be explained by engaging in protective health behaviors. Third, social participation may generate direct physiological benefits, such as buffering stress, enhancing host resistance, and reducing disease risk. It can help older adults obtain important health-related information and maintain self-efficacy for remaining physically or cognitively active. Social participation can stimulate brain functioning in older adults through play, learning, memorizing new information, and practicing new skills [25].

Regarding empirical research on social participation and depression, existing studies have taken four approaches to measuring social participation. The first three were implemented in cross-sectional data. First, some studies have examined the impact of certain forms of social participation on depression incidence, arguing that the strength of the correlation between social participation and depressive symptoms in older adults varies according to the type of social activity. Internet use, sports segments and volunteer participation have all been shown to alleviate depression in older adults. Internet use significantly reduces depression in older adults by increasing social network support [26]. It can also alleviate depressive symptoms by fulfilling basic psychological needs [27]. Sports exercises still have significant effects on reducing depressive scores in older adults [28]. A longitudinal study in the U.S. also showed that volunteer activities involving social participation reduce depression levels among adults older than 65 years [29]. Volunteer service enhances well-being by increasing self-esteem, which is a potent predictor of subclinical depression [30]. Similar findings were obtained in China, where older adults who participated in volunteer services had lower depressive symptoms than did younger adults [31]. Second, some have examined the overall level of social participation in depression, finding that active participation by older adults helps reduce depressive moods [32]. Guided by activity theory, Zhang Chong et al. discovered that older adults with higher levels of participation in social activities also had less depressive moods [33].

Third, some studies have explored the impact of overall patterns of social participation on depression incidence. Li identified types of social participation among older adults and found that simple socialization, intellectual participation, physical exercise, and group organization social participation could significantly reduce the risk of depression in older adults [34]. Social participation can reduce the risk of geriatric depression through mechanisms such as improving mental health, enhancing physical health, and increasing opportunities for individuals to obtain support [35]. Morrow-Howell also described participation patterns of low activity, moderate activity, high activity, productive activity, and fitness activity, revealing that the low-activity group had poorer health than the other groups [36].

The fourth approach was implemented with longitudinal data. Social participation can significantly reduce initial levels of depressive mood in older adults [37, 38]. Regarding trajectories of social participation among older Chinese adults, Xu Jinyan et al. proposed an increasing trend over time, while Ye identified three trajectory types: high declining, moderate declining, and low increasing [39]. However, the latter study had a 50% attrition rate, leading to sample selection bias. Using both cross-sectional and longitudinal data, Glass found independent cross-sectional associations but longitudinal associations only among initially nondepressed individuals in a cohort of community residents aged 65 [40].

### Research in this study

From a life course perspective, social participation fluctuates over time, exposing individuals to varying degrees of social integration during different periods, which may have differential or cumulative impacts on health. With the potential loss of formal social roles, participating in social activities becomes especially important for older adults. Therefore, we used trajectory analysis to capture changes in social participation among older adults, as individual patterns of social participation over time may have significant impacts on their incidence of depression. Based on previous related research, we find a lack of dynamic perspectives in social participation research [41, 42] and limited literature exploring how changes in social participation affect depression in older adults. This study seeks to fill this research gap by tracking changes in social participation trajectories.

First, identifying social participation trajectories over time is a valuable way of verifying activity theory, disengagement theory, and continuity theory from the life course perspective. We need to utilize a more complete sample to explore changing patterns of social participation across multiple timepoints among older Chinese adults, hypothesizing heterogeneity in social participation trajectories: increasing, decreasing, and low-steady trends rather than just a single increasing trend.

Few empirical studies have examined the impacts of changing social participation patterns on depression incidence. Previous research has elucidated the importance of examining changing social participation patterns and proposed their potentially protective effects against depression. Drawing on this evidence and the social integration theoretical framework, this study hypothesizes that older adults with declining social participation levels over time face greater risks of depression.

Therefore, based on the existing research background and results, we propose two research hypotheses:

Based on the three key theories in gerontology that suggest social participation patterns are may shift over time based on personal, social, and environmental factors. Followed the different claims discussed above, I propose hypothesis 1: the social participation of the older adults has three trajectories: increasing, decreasing and steady.

Based on a growing body of empirical research indicating that increased social participation is linked to reduce risk of depression. Studies discussed before have demonstrated that social participation provides emotional support, reduces feelings of loneliness, and promotes psychological well-being, thus mitigating the risk of depression in later life. Therefore, I propose hypothesis 2: The higher level of social participation, the lower risk of depression in the older adults.

## Data and models

### Data and sample

This study utilizes five waves (2011–2020) of tracking survey data from the China Health and Retirement Longitudinal Study (CHARLS), which were released. The CHARLS covers 12,073 households in 150 counties (districts and cities) across 28 provinces in China and has extensive geographic representation. Relevant data were selected from the “Individual Basic Information”, “Health Status and Functioning”, and “Pension and Retirement” modules of the CHARLS database.

The sample screening procedure began with a baseline sample of 17,705 individuals in 2011. Follow-up data was collected in 2013 (*n* = 15,179), 2015 (*n* = 14,574), 2018 (*n* = 13,566), and 2020 (*n* = 13,094), with a total of 16,565 individuals participating in at least one follow-up during the study period between 2011 and 2020.

Several exclusion criteria were applied to refine the sample for the final analysis. Participants were excluded if they were under the age of 60 or had missing data (*n* = 3,143), lacked baseline social participation data (*n* = 7,343), did not have depression measurements at baseline (*n* = 475), or were diagnosed as depressed at baseline (*n* = 3,159).

This resulted in a final sample of 2,445 individuals, contributing a total of 12,225 person-years to the research.

### Variable measurement

#### Social participation trajectories

In the CHARLS questionnaire, respondents reflect their social participation by answering “Have you done any of these social activities in the last month? (Multiple choice)”. Li suggests that older adults engage in social activities during their free time and categorizes these interactions into five types: mutual interaction-based, intellectual activity-oriented, exercise-based, NGO/civil society-related, and help-oriented [35]. These forms foster deeper interactions and tighter bonds with individuals in the community. In line with the study, we incorporate these seven criteria in the survey: “Interacted with friends”, “Played Mahjong, chess, cards, or went to a community club”, “Provided help to family members, friends, or neighbors not living with you”, “Went to a sport, social, or other kind of club”, “Took part in a community-related organization”, “Cared for a sick or disabled adult” and “Attended an educational or training course. If the respondent engaged in an activity in the past month, it was assigned a value of 1; otherwise, it was assigned a value of 0, this measurement also consistent with Zheng [43]. Basis on this, the assigned values for different types of social participation were summed as the final measurement of social participation, with higher scores indicating more participation in that type of social activity in the past month. Trajectories of social participation were based on changes in respondents’ social participation levels over time. The derivation of these trajectories is described in more detail in the analysis strategy section below.

#### Depression risk

Depression was measured using the Center for Epidemiologic Studies-Depression (CES-D) scale in the CHARLS, a commonly used scale for measuring depression internationally. The survey included 10 questions about how the respondent felt and behaved during the past week: Bothered by things that don’t usually bother me, Had trouble keeping my mind on what I was doing, Felt depressed, Everything I did was an effort, Hopeful about the future, Fearful, Sleep was restless, Happy, Felt lonely, Could not get going.

The CES-D score was measured on a 4-point scale, from rarely to some days (1–2 days) or occasionally (3–4 days) to most of the time (5–7 days). We scored these answers using the guidelines suggested by Radloff [44]. Numbers from 0 for rarely to 3 for most of the time were used for negatively worded questions such as “Felt depressed”. For positive questions such as “Happy”, the score was reversed from 0 for most of the time to 3 for rarely. The total score ranged from 0 to 30. A higher score indicated a higher level of depressive symptoms, with a cutoff score of 10 used for depressive symptoms [45]. These were coded as 1, and nondepressed respondents were coded as 0, resulting in a binary variable for depression incidence. Lei measured the validity of this CES-D [46].

The CESD-10 has been extensively employed in previous studies to measure depression among Chinese older adults, with references supporting its usage [43, 47]. In five waves of the Cronbach coefficients of the CES- D were 0.8381, 0.8041, 0.8032, 0.8093 and 0.8203, indicating a reasonable level of internal consistency.

#### Control variables

This study controlled for sociodemographic characteristics, health status, and living standards, variables commonly recognized in the literature as potential confounders in studies of social participation. Sociodemographic characteristics included age (in years), sex (1 = female, 0 = male), and marital status (1 = married, 0 = unmarried) [48]. Health status was measured by IADL scale scores, covering abilities in cooking, housework, medication management, telephoning, shopping, and financial management across 6 items. Patients who were difficult but still able to complete the questionnaire, had difficulty and needed assistance, and were unable to complete any of the 6 items were defined as having disabilities. Respondents were evaluated as disabled if they had disabilities according to any of the 6 indicators [49]. Due to extensive missing values in income measurement, we used responses such as “Overall, how do you rate your standard of living?” to measure living standards. This question asked participants to rate their standard of living as ‘very high’, ‘relatively high’, ‘average’, ‘relatively poor’, or ‘poor’, with higher scores indicating greater wealth [48].

### Analytical approach

This study employed growth mixture modeling (GMM) to estimate trajectory classes of social participation over time. Categories derived from the GMM analysis of social participation trajectories were subsequently used as a set of dummy variables in Cox proportional hazards models to examine relationships between these social participation trajectory categories and depression incidence rates. Analyses were conducted using Mplus [50]and Stata. Growth mixture modeling has been widely applied in various types of trajectory research.

The GMM combines features of conventional growth modeling and latent class growth analysis (LCGA). Conventional growth modeling assumes that all sample individuals come from a single population and estimates one average growth curve. Individual variation around the average growth curve is captured by estimating growth factor variances. The LGA estimates an average growth curve for each class. No individual variation around the class average growth curves was allowed. Thus, the growth factor variances within each class are assumed to be zero. The GMM estimates average growth curves for each class and captures individual variation around these growth curves by estimating growth factor variances separately for each class. The following equations apply for a linear growth mixture model with K latent trajectory classes, where in class k (k = 1,2,… K):

1 $$\:{y}_{it}=\:{\eta\:}_{0i}+{\eta\:}_{1i}{\alpha\:}_{kt}+{\epsilon\:}_{it}$$

2a $$\:{\eta\:}_{0i}=\:{\alpha\:}_{0k}+{\gamma\:}_{0k}{w}_{i}+{\zeta\:}_{0i}$$

2b $$\:{\eta\:}_{1i}=\:{\alpha\:}_{1k}+{\gamma\:}_{1k}{w}_{i}+{\zeta\:}_{1i}$$

Equation 1 represents within-individual change over time. Equation 2a and 2b represent between-individual changes over time. The outcome variable (i.e. social participation level) is $\:{y}_{it}$, $\:{\eta\:}_{0}$the intercept, $\:{\eta\:}_{1}$ is the slope, t is the timepoint, and w is a covariate. The subscript i denotes parameters varying across individuals. The $\:{\alpha\:}_{k}$ parameters vary across classes to capture different trajectory types. The $\:{\gamma\:}_{k}$ parameters allow covariate effects on growth factors to differ across classes. The residuals are denoted by $\:{\epsilon\:}_{it}$、$\:{\zeta\:}_{0i}$ and $\:{\zeta\:}_{1i}$.

Incorporating covariates is important when determining the number of classes in a growth mixture model. Thus, age, sex, marital status, living standards, and education were included in the modeling process of determining the number of trajectory classes. The optimal model had relatively small Bayesian information criterion (BIC), sample-size adjusted BIC, and Akaike information criterion (AIC) values, as well as significant Lo, Mendell, and Rubin [51] likelihood ratio test (LMR-LRT) statistics [52]. The entropy index was used to evaluate the classification accuracy and ranged from 0 to 1. An entropy = 0.6 indicates approximately 20% misclassification, while an entropy of approximately 0.8 indicates a classification accuracy greater than 90% [53].

Once social participation trajectory categories were derived from the growth mixture model, these categories were coded into a set of dummy variables and entered into Cox proportional hazards models to examine relationships between changing social participation patterns over time and depression incidence rates. The proportional hazards assumption was verified. Although growth mixture modeling allows individual variation around each growth curve, in this study, individuals were treated as fixed in their best matching trajectory category, eliminating individual variation when individuals were coded as dummy variables for the Cox proportional hazards models. The analysis sample included 2,445 person and 12,225 person-years.

## Results

### Descriptive analysis

**Table 1:** Descriptive statistics (mean or percentage)

Variables	2011	2013	2015	2018	2020
age	68.12				
male	60.41%				
urban	28.26%				
education					
illiteracy	28.34%				
primary	46.34%				
middle above	25.28				
married	72.47%	66.30%	60.04%	50.31%	45.40%
disable	16.52%	18.12%	19.43%	23.27%	22.41%
living standards	2.61 (0.71)				

Data source: Calculated based on 2011–2020 China Health and Retirement Longitudinal Survey (CHARLS) data. Unless otherwise specified, the data source for the following charts is the same as Table 1

Table 1 displays the descriptive statistics of the variables. At baseline, the respondents had a mean age of 68.12 years. Over half of the participants had an average education level of primary school or below. The self-rated living standards were generally medium. Most of the respondents were male (60.41%). At baseline, 72.47% of the respondents were married. The prevalence of functional impairments in IADLs among respondents increased annually from 16.52 to 22.41%.

Therefore, our sample primarily consisted of older adults, with most participants living in rural areas. They generally had lower education levels, experiencing an increase in disability and losing their spouses over time.

### Social participation trajectories

**Table 2:** Summary of GMM model fit information

model	K	Log(L)	AIC	aBIC	Entroy	LMR	BLRT	class probability
1	7	-14,820	29654.623	29695.235	-	-	-	-
2	10	-13,963	27947.413	28005.431	0.843	0.000	0.000	0.15/0.85
**3**	**13**	**-13,718**	**27463.776**	**27539.199**	**0.828**	**0.000**	**0.000**	**0.20/0.75/0.05**
4	16	-13,607	27247.341	27340.169	0.817	0.051	0.101	0.06/0.73/0.03/0.17

**Fig. 1:**
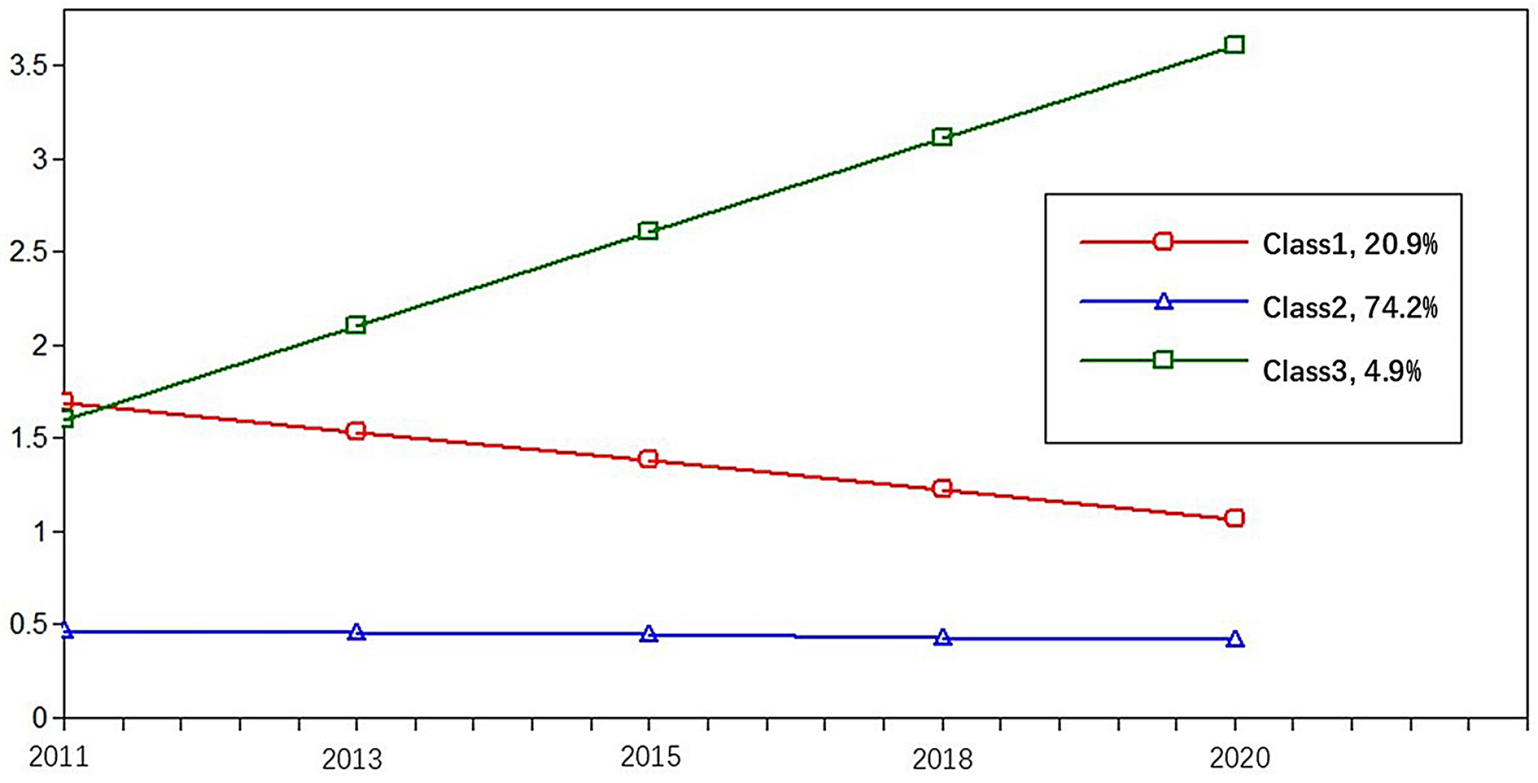
Estimated mean values of social participation trajectory classes

**Fig. 2:**
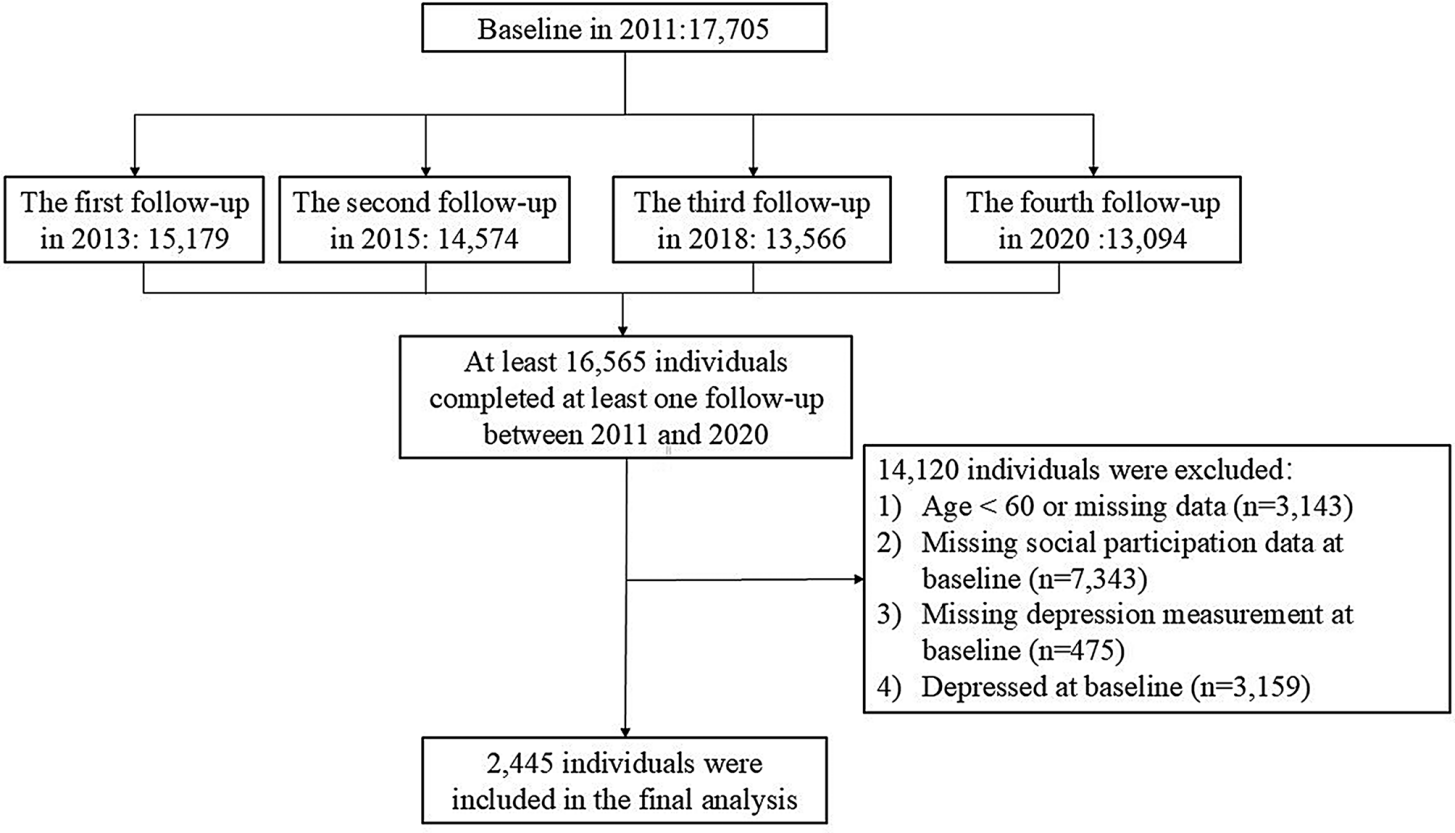
The sample screening procedure

Table 2 and Fig. 1 present the social participation trajectory classes. A three-class solution for the social participation trajectories provided the best fit for the growth mixture model data. This model had smaller information criterion values (AIC and sample-size adjusted BIC) than did the other class solutions and had significant LMR and BLRT test statistics. It had a high entropy of 0.854, indicating almost no overlap in the clear delineation of classes. By further analyzing the characteristic trends of each social participation trajectory class, based on the three types provided in Fig. 2, we categorized them as decreasing, low-steady, or increasing according to the tendencies of the trajectories.

Therefore, three social participation trajectories were identified: increasing (4.9%), low-steady (74.2%), and decreasing (20.9%). The increasing trajectory represents individuals whose social participation began at a moderate level and gradually increased over time. The decreasing trajectory, in contrast, does not correspond to a low level of social participation; rather, it reflects a moderate level of participation with a slight downward trend over time, indicating a reduction in social participation but still maintaining a relatively higher level of participation compared to the low-steady trajectory. The low-steady trajectory, on the other hand, represents individuals with consistently low levels of social participation throughout the study period.

### Social participation trajectories and the risk of depression

**Table 3:** Hazard ratios for the relationship between social participation trajectories and depression risk

Variables	Model 1	Model 2
**decreasing**	0.81** [0.70, 0.93]	0.84** [0.73, 0.97]
**increasing**	0.76** [0.56,** 0.98]**	0.79 [0.60, 1.05]
age		1.03 [0.98, 0.99]
education		0.95** [0.89, 0.99]
non-rural		0.75*** [0.68, 0.82]
men		0.73*** [0.68, 0.78]
having a spouse		0.92 [0.85, 0.99]
non-disabled		0.58*** [0.54, 0.62]
living standards		0.84*** [0.81, 0.88]
Sample num.	12,225	10,403

Note: low-steady is a reference; *p <.05. **p <.01. ***p <.001.; Values in parentheses represent the 95% confidence intervals

The social participation trajectory categories were entered into Cox proportional hazards models as dummy variables to examine the relationships between the social participation patterns and depression incidence rates (shown in Table 3). The low-steady social participation trajectory was the reference category.

In the model with only social participation trajectory types (Model 1), compared to those in the low-steady trajectory, belonging to the decreasing social participation trajectory was associated with a significantly lower depression risk by 19%. Besides, the decreasing trajectory has a higher level of social participation than the low-steady trajectory. Moreover, compared to those in the low-steady trajectory, belonging to the increasing social participation trajectory was associated with a significantly lower depression risk by 24%. Besides, the increasing trajectory has the highest level of social participation.

Model 2 included sociodemographic variables as controls in Model 1. Belonging to the decreasing social participation trajectory was significantly associated with lower depression risk, with a 16% lower depression risk for those in the low-steady social participation trajectory. Although the increasing trajectory was also associated with a lower depression risk than was the decreasing trajectory, this difference was not statistically significant because of the smaller sample size of the increasing trajectory.

Due to missing data in some control variables, the complete case analysis resulted in different sample sizes in different models. We examined the missing patterns and performed robustness analyses, and the results showed that the main conclusions remained consistent, indicating that the impact of missing data was within an acceptable range.

Therefore, compared to the low-steady trajectory with no social participation, the decreasing trajectory with higher levels of participation was associated with lower rates of depression.

### Conclusion and discussion

This study used social integration theory to examine the relationships between changing patterns of social participation and depression incidence rates under the guidance of activity theory, disengagement theory, and continuity theory within the life course framework. We found three types of social participation trajectory developments among older Chinese adults: increasing, decreasing, and low-steady. Compared to the other two trajectories, the declining social participation trajectory increased depression risk. This result verified our hypothesis, indicating the importance of social integration and social embeddedness achieved through social participation for depression risk. However, this protection may depend on maintaining certain levels rather than low steady social participation over time.

From the life course perspective, confirming hypothesis 1, we fitted three types of social participation trajectories—increasing, decreasing, and low-steady—which supported the three assertions on social participation in gerontology theories: activity theory, disengagement theory, and continuity theory. However, considering the proportions of individuals in each trajectory type, older adults with decreasing and low-steady trajectories accounted for a relatively large share of the population, while the share of older adults with increasing trajectories was quite small.

Compared to previous studies, our research identifies both similarities and differences in older adults’ social participation trajectories. Similar to Zhou’s study [54], which found diverse participation patterns, our results also highlight varied trajectories. However, the three types we identified—increasing (4.9%), low-steady (20.9%), and decreasing (74.2%)—differ from Zhou’s three patterns: slow decline, slow increase, and mid-life stability. Notably, our study found a smaller proportion of older adults in the increasing trajectory, whereas Zhou’s research showed that nearly half of participants were in the slow increase and mid-life stability groups. Additionally, our low-steady group (20.9%) reflects that most older adults maintain a relatively low level of participation—a pattern not clearly identified in Zhou’s study. These differences may be attributed to two factors. First, Zhou included household chores as social participation, while our study focused on interactive social engagement, excluding household chores. Second, Zhou’s sample consisted of younger older adults, who may be more likely to engage in social activities.

Our findings align more closely with Zhang’s study [55], which identified four trajectories: stable, slow decline, low-scoring with decline, and high-scoring with decline. Over half of Zhang’s sample showed rapid decline, consistent with our results. Additionally, only a small proportion of older adults exhibited increasing participation, supporting previous research that suggests most older adults disengage as they age [19]. The increasing trajectory in our study is also consistent with findings from Ye [56] and Xu [57]. Ye identified a low-growth trajectory, while Xu found that 41.5% of older adults maintained a high baseline level of participation, which continuously increased.

The disparities in social participation trajectories suggest that, from a life course perspective, older adults’ social participation is better explained by disengagement and continuity theories. This contrasts with the widespread use of activity theory in previous studies, where older adults were often labeled as “active” or “positive.” Such labels may reflect cross-sectional differences at a single time point, rather than changes over time. In contrast, disengagement and continuity theories more accurately capture the temporal changes in social participation, indicating that physical decline or other constraints can reduce one’s ability to engage in social activities, leading to decreased participation [20].

The results differ from a study conducted in the USA, which identified five distinct social participation trajectories among older adults [58]. Cultural differences may account for this variation, as China’s extended family structure may impose more constraints on social participation compared to the more individualized family structures in Western societies [59]. In traditional Chinese families, the parent-child relationship follows a “feedback model,” where parents are responsible for nurturing their children, and children, in turn, care for aging parents [60]. As aging increases and the three-child policy leads to more multi-generational households, the rising need to care for both elderly parents and young grandchildren creates a dual caregiving burden for many older adults. As a result, older individuals have fewer social interactions, and their participation patterns are often more passive. This helps explain the low proportion of increasing trajectories in our study.

From the perspective of social integration, this study confirms hypothesis 2, which suggests that older adults in the low-steady trajectory of social participation face a higher risk of depression compared to those in the increasing or decreasing trajectories, who exhibit a lower risk, which is consisted with the previous studies. As reduced participation often leads to social isolation, which is detrimental to health. Social isolation has been associated with a 2–4 times higher mortality risk compared to those with strong social ties [53, 54]. Reduced social participation reflects diminished social integration, and since social participation is crucial for maintaining integration, it negatively impacts mental health, as outlined by social integration theory [55]. Meanwhile, this study identified low level trajectory of social participation can lead high risk of depression. The result was consistent with previous study, which also found low social participation may increase the risks of depression [61], also consistent with Wang’s study, which found lower social activity participation significantly negatively affected the increase of depressive symptoms in old age at that time [62]. In comparison to studies in China, our research aligns with Li’s approach to measuring social participation, but we expand upon it by adopting a longitudinal perspective. This extension further confirms that social participation significantly reduces the risk of depression among older adults [35]. Social participation enhances social trust, which in turn plays a crucial role in reducing the risk of depression among older adults [63]. Beyond its individual benefits, social participation may also contribute to mental well-being at a broader societal level by promoting social equity, thereby helping to alleviate depression on a larger scale [64].

Moreover, the results of several significant control variables indicate that increasing age is a risk factor for depression, while living in urban areas, being male, having a higher standard of living, and not being disabled are protective factors. These findings are consistent with previous research [3].

This study’s contributions include the following.

First, compared to previous studies on social participation trajectories, this research is the first to identify an increasing trajectory, alongside the decreasing and steady trajectories, at the theoretical level, this study verifies activity theory. This study uses disengagement and continuity theories to examine older adults’ social participation trajectories from a life course perspective. It critiques the common reliance on activity theory in analyzing the social participation-health relationship using cross-sectional data, which overlooks the theory’s emphasis on temporal change. While high social participation at a single point may correlate with better health, this static approach fails to capture the dynamic nature of social participation over time, as highlighted by gerontological theories. Additionally, incorporating social integration theory within the life course framework deepens our understanding of its link to health. The life course perspective emphasizes both stability and change in aging, highlighting the evolving nature of social integration, which is often overlooked in existing research.

Second, this study contributes further measurements of social participation to the literature. Most of the existing research has measured overall levels of social participation in cross-sectional data or utilized latent class analysis to determine different patterns of social participation [63], reflecting older adults’ social participation status at a certain point. Our research provides a new perspective by discussing changes in social participation among older adults over time using longitudinal data, enriching the existing research findings.

Third, there has always been debate around two-way causality in the social participation-health relationship [65–67]. To address this issue to a certain extent, we utilized 10 years of complete tracking follow-up data on the same sample, reducing the influence of individual variation. We also used a sample without attrition, lowering the impacts of sample bias on the results. Additionally, samples from patients with depression at baseline were not included. Health factors affecting individuals’ ability to engage in daily activities, which mainly influence opportunities for social participation, were controlled for. Under these circumstances, declining social participation trajectories remained a strong significant predictor of depression incidence rates, providing robust evidence for the benefits of social participation on depression risk.

Based on the results of this study, decreasing social participation trajectories demonstrated significantly declining social participation with increasing age, while the impacts of social participation on health conditions increased with age. There is a need to develop effective interventions targeting these declining trends in social participation to prevent, delay, or even potentially reverse adverse health consequences. Social participation accumulates over time, forming different cumulative patterns. Early-life social participation patterns have primary impacts on later-life patterns. Therefore, emphasizing social participation at younger ages is essential for promoting social participation from a life course continuity perspective [16].

However, our study has several limitations. First, it did not account for the quality of social participation. Social interaction quality can vary, and negative experiences may affect health differently than positive ones. Second, selective survival may introduce bias, as participants lived for 60 years, potentially skewing results. Third, the study fixed trajectory classifications, overlooking individual variability. Future research should address these aspects. Forth, this study treated living standards as a time-invariant variable, using only baseline data. Since living standards may change over time, ignoring its temporal variation could introduce bias in effect estimates. Future research could incorporate longitudinal data to better assess its impact. Lastly, the sample was primarily composed of older adults from rural areas with lower education levels, increasing disability, and higher rates of spouse loss. As such, the findings may be more relevant to this demographic and may not fully represent the broader older adult population in China. Caution is therefore needed when generalizing these results.

To promote positive social participation, two key measures can be taken. First, improving community infrastructure is crucial, including increased investment in activity centers, senior universities, and fitness facilities, alongside enhanced accessibility to ensure older adults can easily engage in community life. Second, expanding childcare and elderly care services can alleviate caregiving burdens on older adults. More childcare centers and day-care facilities would free up their time and energy for social participation. It is essential that a financial support system be established to provide subsidies to families with significant caregiving responsibilities, particularly those facing economic hardship.

Despite its limitations, this study provides valuable theoretical frameworks for exploring the relationship between social participation and depression. By integrating activity, disengagement, and continuity theories within a life course perspective, the study enriched the analysis of longitudinal data. The findings highlight the risks associated with declining social participation and demonstrate that higher levels of social engagement are linked to a reduced risk of depression. Maintaining high social participation over time is therefore an effective strategy for promoting mental health among older adults.

## Data Availability

CHARLS data is available through individual user registration. All details about the application and registration process can be found at https://charls.pku.edu.cn/.
